# Supplementary material for: Chromothripsis during telomere crisis is independent of NHEJ, and consistent with a replicative origin
Source: Genome Res. 2019 May;29(5):737–49. doi: 10.1101/gr.240705.118 (PMC6499312; doi:10.1101/gr.240705.118)
Supplement: Supplemental Material [file supp_gr.240705.118_Supplemental_file_1.zip › contigs/annotated_contigs/DB41/contig.2.DB41_length_302_mean_cov_4.96688741722.docx]

**DB41_length_302_mean_cov_4.96688741722**

```
AATTCAAGGTGAGATTTGGGTAGGGACACAGACCCAAACCATATCACCAGGAAAATAAAATCCTTTAGCATTCATGACTGTCAATGCTT
 >chr5:25596848-25596978 + E=6e-67 p=4e-02
AACATTGTGCTGGCTGGTAAAATTATATATATATATATATA|ATATATAATATAATC|CTGTGGGATTTTCAAGGCAGTAGAGAAGCAA
                                                         >chrX:3413627-3413784 - E=3e-8
GCCTTGAAAGCAACAATGAATGAGAAATATTCTCCATTTTTTATGTGGGTTGTTGGATCCATGCTTATTTTATTGGCTTGCTTCTTAAC
3
ATGCTTTGCATATTCATCCTTTCACACCATTGCATAT
```
